# Biomarkers of post-discharge mortality among children with complicated severe acute malnutrition

**DOI:** 10.1038/s41598-019-42436-y

**Published:** 2019-04-12

**Authors:** James M. Njunge, Agnes Gwela, Nelson K. Kibinge, Moses Ngari, Lydia Nyamako, Emily Nyatichi, Johnstone Thitiri, Gerard Bryan Gonzales, Robert H. J. Bandsma, Judd L. Walson, Evelyn N. Gitau, James A. Berkley

**Affiliations:** 1 The Childhood Acute Illness & Nutrition (CHAIN) Network, Nairobi, Kenya; 2 KEMRI/Wellcome Trust Research Programme, Kilifi, Kenya; 3 Department of Gastroenterology, Faculty of Medicine and Health Sciences, Ghent University, Ghent, Belgium; 4 African Population and Health Research Centre, Nairobi, Kenya; 5 Centre for Tropical Medicine & Global Health, Nuffield Department of Medicine, University of Oxford, Oxford, UK; 6 Departments of Global Health, Medicine, Paediatrics and Epidemiology, University of Washington, Seattle, Washington USA; 7 Centre for Global Child Health, The Hospital for Sick Children, Toronto, Ontario Canada; 8 Inflammation Research Centre, Flemish Institute for Biotechnology, Ghent, Belgium

## Abstract

High mortality after discharge from hospital following acute illness has been observed among children with Severe Acute Malnutrition (SAM). However, mechanisms that may be amenable to intervention to reduce risk are unknown. We performed a nested case-control study among HIV-uninfected children aged 2–59 months treated for complicated SAM according to WHO recommendations at four Kenyan hospitals. Blood was drawn from 1778 children when clinically judged stable before discharge from hospital. Cases were children who died within 60 days. Controls were randomly selected children who survived for one year without readmission to hospital. Untargeted proteomics, total protein, cytokines and chemokines, and leptin were assayed in plasma and corresponding biological processes determined. Among 121 cases and 120 controls, increased levels of calprotectin, von Willebrand factor, angiotensinogen, IL8, IL15, IP10, TNFα, and decreased levels of leptin, heparin cofactor 2, and serum paraoxonase were associated with mortality after adjusting for possible confounders. Acute phase responses, cellular responses to lipopolysaccharide, neutrophil responses to bacteria, and endothelial responses were enriched among cases. Among apparently clinically stable children with SAM, a sepsis-like profile is associated with subsequent death. This may be due to ongoing bacterial infection, translocated bacterial products or deranged immune response during nutritional recovery.

## Introduction

Malnutrition is an underlying cause of nearly half of global deaths in children below 5 years mainly in sub-Saharan Africa and Asia^1,2^. Evidence suggests that most deaths are due to common infectious diseases, mainly pneumonia, diarrhoea, and malaria; and lower nutritional status is associated with markedly increased risk of mortality across these syndromes^1–7^. Children with severe acute malnutrition (SAM) are treated according to standardized World Health Organization (WHO) guidelines using antibiotics, correction of nutritional deficiencies through therapeutic feeding, and treatment of other medical conditions^8^. Children with complicated SAM defined as those with infections, severe oedema, failing an appetite test, or presenting with one or more Integrated Management of Childhood Illness (IMCI) danger signs are initially treated as inpatients before being discharged to outpatient therapeutic feeding care once stabilized^8^. Complicated SAM is typically associated with an in-patient mortality risk of 12% to more than 30% in African hospitals^9–12^.

A high rate of mortality in the months following hospital discharge has been observed among children with SAM in sub-Saharan Africa^13–22^. A recent systematic review reported paediatric post-discharge mortality rates in resource-poor countries of up to 18% which may exceed in-hospital mortality rates in many settings^18,23^. This implies that processes underlying susceptibility to mortality continue beyond the clinically evident acute episode. In these studies, poor nutritional status, young age, and HIV were all associated with higher mortality risk post-discharge. However, while anthropometry and clinical parameters are important in selection of children requiring specialized care and monitoring, they are non-specific and do not indicate specific mechanisms that may lead to death. Whilst mechanisms resulting in post-discharge deaths are not well understood, they are likely to involve the interaction between the metabolic consequences of malnutrition, infection, and intestinal dysfunction, whose causes and consequences are “cyclic” and self-reinforcing^7,24^. This sustained risk of death post-discharge following apparent medical stabilization calls for an understanding of processes that could be amenable to intervention.

The objective of this study was to characterize the plasma proteomic profile associated with early post-discharge mortality in children who were medically treated for complicated SAM and nutritionally stabilized. We hypothesized that early post-discharge mortality in children with SAM is associated with derangements in certain biological processes that could be ascertained by measuring plasma proteins measured before discharge from the hospital. Specifically, we hypothesized that an increased inflammatory response is associated with mortality. Hence, we used both untargeted (plasma protein profiles) and targeted (cytokines, chemokines, and leptin) approaches to determine associations with mortality within the next 60 days among children who had been judged medically stabilized according to WHO guidelines.

## Results

### Characteristics of study children, clinical causes of mortality, and full blood count

The baseline characteristics of the study children measured at discharge are given in Table 1. Children (n = 241) in this nested case-control study had mean MUAC of 10.4 (SD ± 1.2) and 41 (18%) had nutritional oedema. Post-discharge deaths occurred a median of 19 (Interquartile range, IQR 7–34) days after sampling, mostly (60%) during readmission to the study hospitals, while the rest were in the community (31%) and other hospitals (8%) (Table 2). Pneumonia, sepsis, diarrhoea, and febrile illness were the commonest clinical syndromes recorded as causing death. Blood culture was performed for those readmitted to study hospitals, but few were positive (Table 2). Among the four blood culture isolates identified, only one was likely to be sensitive to one of the recommended first line antibiotics for use in hospital (penicillin or ampicillin plus gentamicin) (Table S3).

**Table 1:** Baseline characteristics of the study participants.

Characteristic	Cases (N = 121)	Controls (N = 120)	*P*	*Padj*
Gender (female) – No. (%)	59 (49)	53 (44)	0.48	—
Age (months) – median (IQR)	9 (5–14)	11.5 (7–16)	0.02	—
Under six months) – No. (%)	34 (28)	13 (11)	0.001	—
***Recruitment site***				
Kilifi – no. (%)	5 (4.1)	6 (5.0)	0.98	—
Mombasa – no. (%)	64 (53)	64 (53)		
Malindi – no. (%)	23 (19)	23 (19)		
Mbagathi – no. (%)	29 (24)	27 (23)		
***Clinical Variables***				
Nutritional oedema – no. (%)	20 (17)	21 (18)	0.84	0.84
Mid upper arm circumference – cm ± sd	10.1 ± 1.2	10.8 ± 1.1	<0.001	0.005
Length-for-age z score ± sd	−3.0 ± 2.0	−2.9 ± 1.7	0.78	0.84
***Diagnosis at index admission***				
Severe pneumonia – no. (%)	49 (41)	40 (33)	0.25	0.86
Diarrhoea – no. (%)	68 (56)	66 (55)	0.85	0.99
Shock – no. (%)	12 (9.9)	12 (10)	0.98	0.99
Clinical signs of rickets — no. (%)	24 (20)	12 (10)	0.03	0.21
Known tuberculosis – no. (%)	7 (5.8)	7 (5.8)	0.99	0.99
Cerebral palsy – no. (%)	4 (3.3)	4 (3.3)	0.99	0.99
Randomized to co-trimoxazole prophylaxis – no. (%)	60 (50)	60 (50)	0.95	0.99
***Full blood count***				
Haemoglobin g/dl ± sd	9.6 ± 2.1	9.8 ± 1.9	0.43	0.57
Platelets counts (×10^3^/L) – median (IQR)	387 (203–531)	474 (280–583)	0.02	0.1
WBC counts (×10^3^/L) – median (IQR)	10.5 (7.9–14.8)	9.3 (6.5–12.8)	0.06	0.1
Lymphocytes counts (×10^3^/L) – median (IQR)	4.7 (3–7.0)	4.9 (2.9–6.7)	0.90	0.9
Neutrophils counts (×10^3^/L) – median (IQR)	3.5 (2.2–5.9)	3.0 (1.9–4.5)	0.05	0.1

IQR = interquartile range, sd = Standard deviation, no = number, *Padj* = P value corrected for false discovery.

**Table 2:** Details of mortality and clinical causes among cases.

Characteristic	Cases
***Mortality***	
Mortality involving first SAE – no. (%)	111 (92)
Mortality involving second SAE – no. (%)	10 (8)
***Location of death***	
Study hospital – no. (%)	73 (60)
Community – no. (%)	30 (31)
Other hospital – no. (%)	10 (8)
***Time to death***	
Days – median (IQR)	19 (7–34)
Clinically assigned cause of death*	
Pneumonia – no.	42
Sepsis – no.	30
Gastroenteritis – no.	29
Unknown (no cause assigned) – no.	24
Unknown febrile illness – no.	17
Encephalopathy of unknown cause – no.	6
Pulmonary TB – no.	2
Malaria – no.	2
***Bacterial pathogens detected in blood***	
*Klebsiella pneumoniae –* no.	1
*Pseudomonas aeruginosa –* no.	1
Non-typhoidal Salmonella sp. – no.	1
*Streptococcus pneumoniae –* no.	1

SAE = severe adverse event, *More than one cause may be present in individuals. no. = number.

Compared to controls, cases were younger (*P* = 0.02) representing a greater percentage of those below 6 months (28% vs 11%, *P* = 0.001). Cases also had significantly lower MUAC (*P* < 0.001) and weight-for-age Z-score (WAZ) compared to controls (*P* = 0.01). Lower platelet counts and higher neutrophil counts were found in cases compared to controls on univariate analysis, however, these were not significant when adjusted for age, site and randomization arm.

### Cases are characterized by low total plasma protein

**Figure 1:**
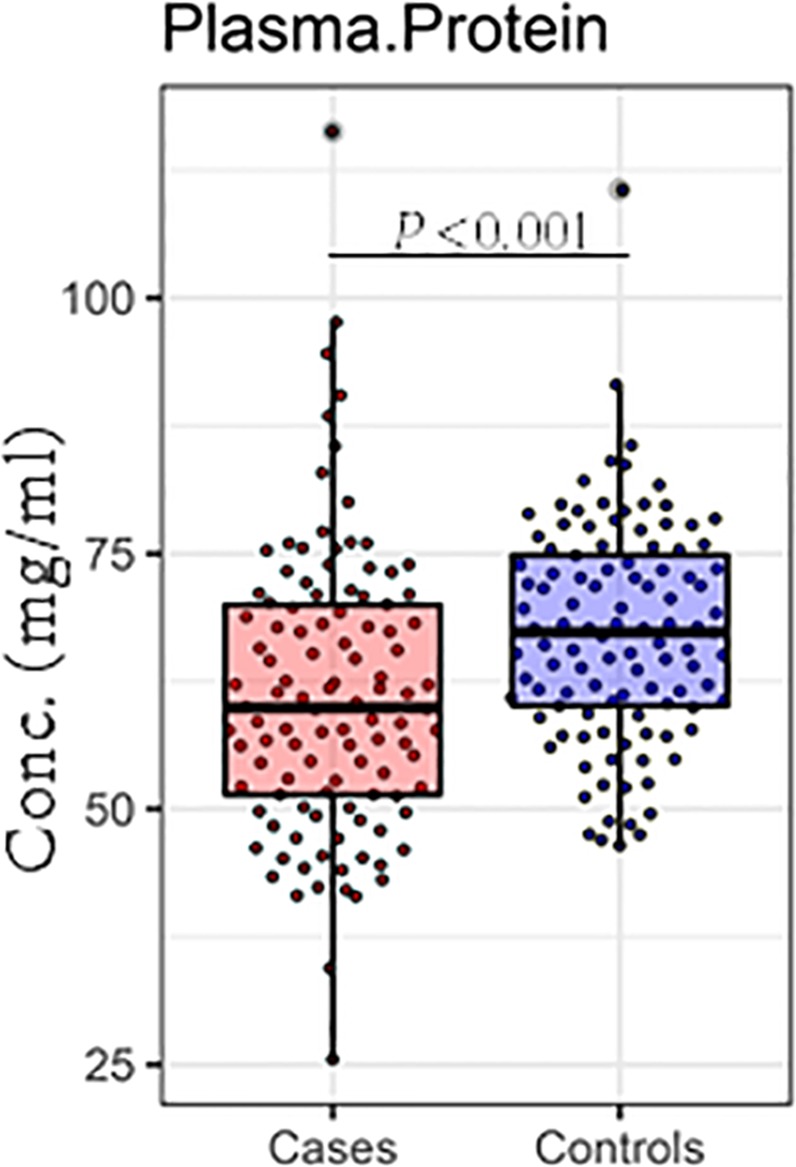
Plasma total protein concentration is significantly different between cases (n = 119) and controls (n = 119). Box plot summarize the median and interquartile ranges of plasma total protein and *P* values < 0.05 are significant.

The plasma total protein is predominantly comprised of albumin and immunoglobulins (reference ranges between 60–80 mg/ml^25–27^). Cases had significantly lower total plasma protein concentration (median; 59.9 mg/ml, IQR; 51.4–70) compared to controls (median; 67.3 mg/ml, IQR 60.1–74.8) (*P* < 0.001, Fig. 1, Table S4). The median total plasma protein among controls was well within the normal reference range and just below the lower threshold of the reference range for cases.

### Systemic inflammatory proteins are increased among cases

**Figure 2:**
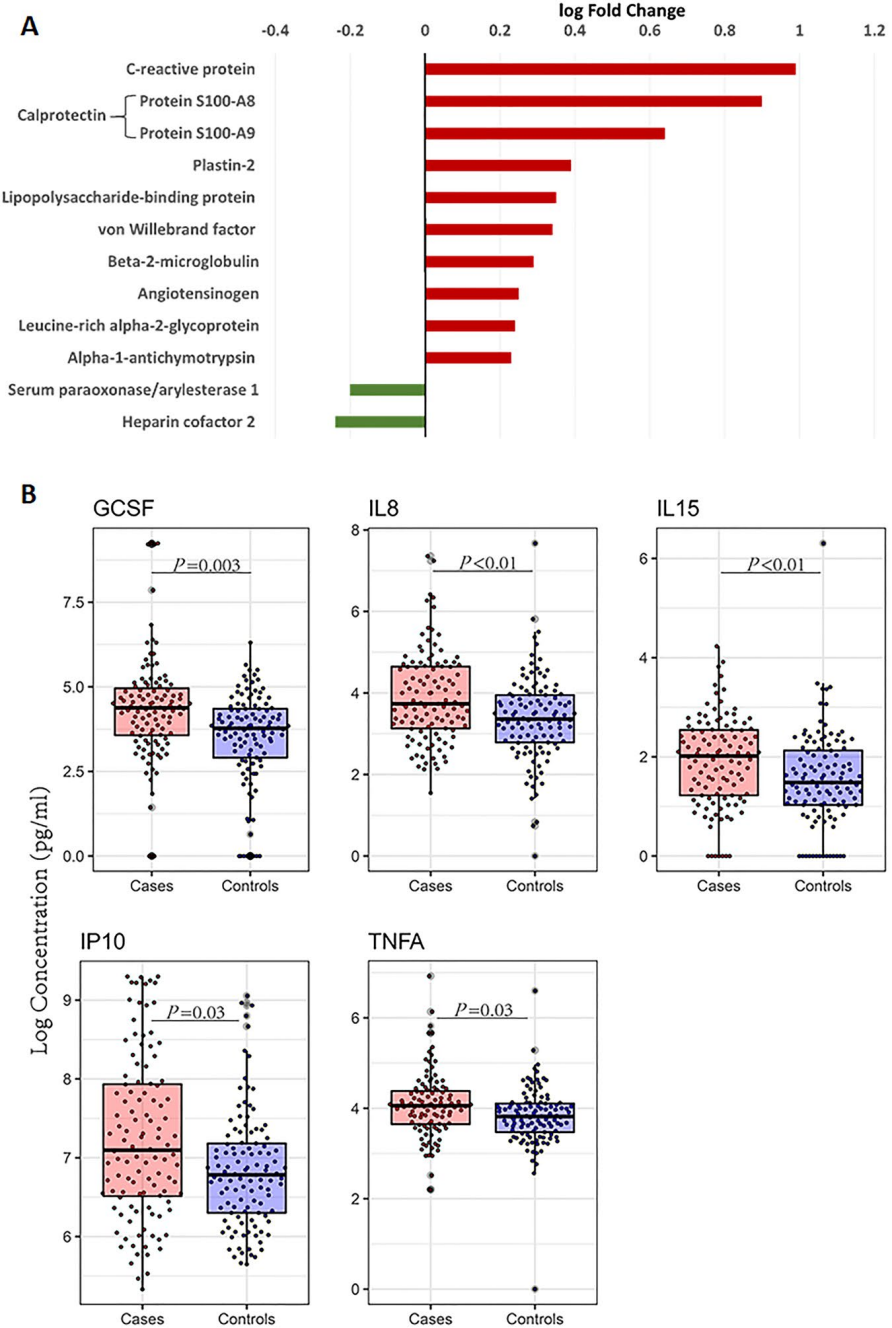
Systemic inflammatory proteins are increased among cases. (**A**) Bar plot of differentially expressed proteins from the mass spectrometry-based proteome analysis between cases (n = 121) and controls (n = 120). The analysis was performed using proteome measurements in plasma among cases and controls. The bar graph depicts the log10 of the fold change of differentially expressed proteins. Red and green bars indicate significantly up- and downregulated proteins among cases. (**B**) Box plot summaries of the median and interquartile ranges of natural logarithm concentrations of 5 cytokines that were significantly different between cases (n = 112) and controls (n = 113). Overlaid dots represent individual data points. GCSF, granulocyte-colony stimulating factor; IL8, interleukin 8; IL15, interleukin 15; IP10, Interferon gamma-induced protein 10 (IP-10)/chemokine (C-X-C motif) ligand 10; TNFA, tumour necrosis factor; P values represent the adjusted *P* value using the Benjamini and Hochberg (BH) method for multiple testing.

We compared expression levels of quantified specific proteins between cases and controls. Our untargeted liquid chromatography-tandem mass spectrometry based proteomics analysis resulted in the relative quantification of 444 proteins after excluding potential contaminants and identifications from the decoy database. A decoy FASTA database is generated from the target database, comprising sequences derived from the organism being studied, by switching the amino-carboxyl orientation of a protein’s amino acids to generate sequences that do not exist in nature, which are then concatenated with the target FASTA database^28^. Of the quantified proteins, 146 (33%) were quantified in all the study children. Twelve proteins were found to be differentially expressed (FDR ≤ 0.05; Fig. 2, Table S5). C-reactive protein, subunits of calprotectin (Protein S100-A8/S100-A9), plastin-2, angiotensinogen, and lipopolysaccharide-binding protein (LBP) were higher among cases, while heparin cofactor 2 and serum paraoxonase/arylesterase 1 proteins were higher among controls (Fig. 2A, Table S5).

Among the cytokines and chemokines assayed (n = 29); IL8, IL15, G-CSF, IP10, and TNFα were significantly higher among cases (FDR ≤ 0.05; Fig. 2B, Table S6). However, sCD14 was not significantly different between the cases and controls (*P* = 0.09, Table S4).

**Figure 3:**
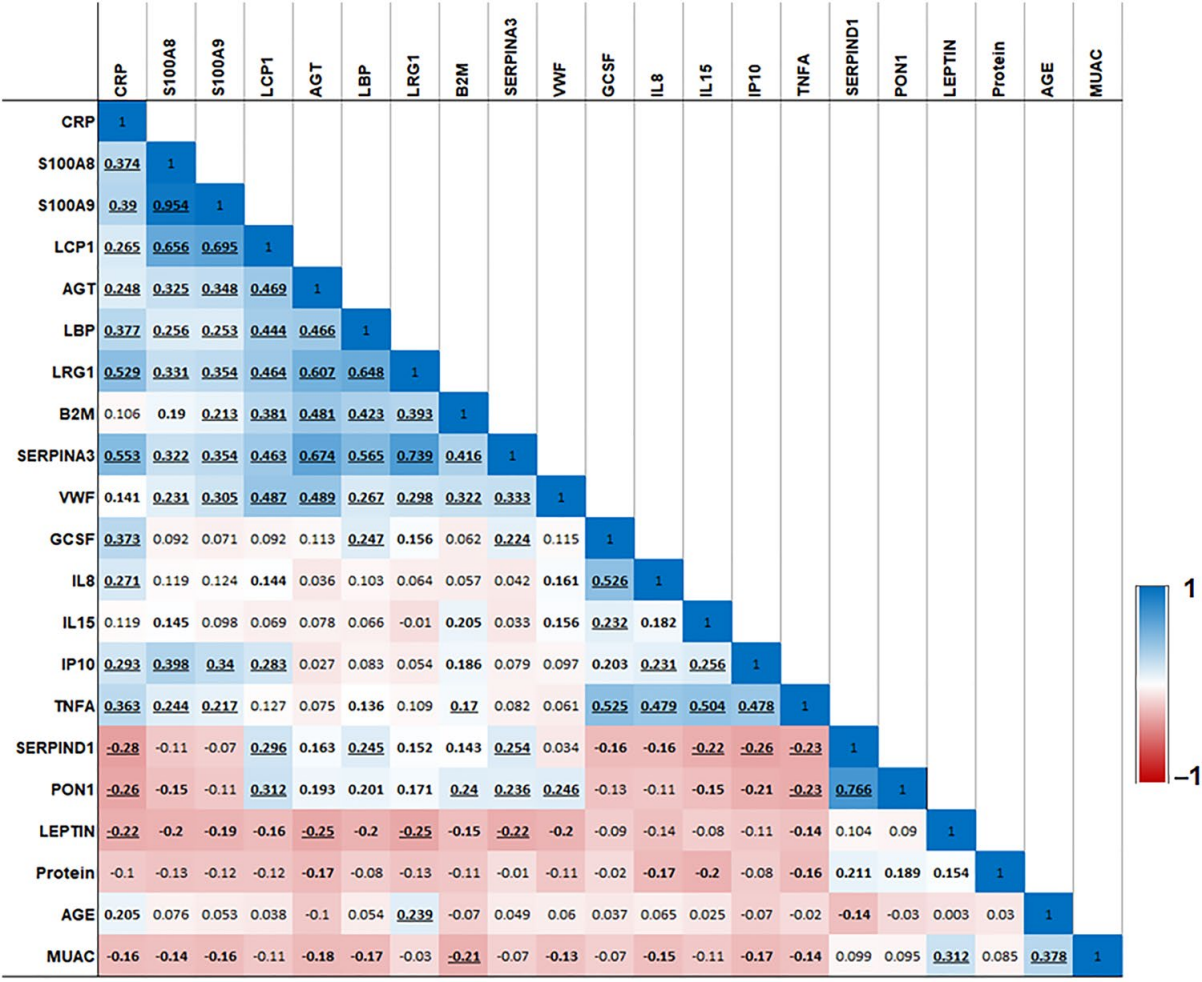
Pairwise correlation heatmap among differentially expressed proteins FDR < 0.05, MUAC, age, Protein, and leptin. MUAC, Mid upper arm circumference. Protein, Total plasma protein. 1 and −1 depicts the highest positive and negative correlation values respectively in the correlation matrix. Pairwise correlations highlighted in bold are *P* < 0.05 and those with bold and underlined are *P* < 0.001.

In pairwise correlation analysis, most inflammatory proteins were positively correlated with each other and with VWF (Fig. 3). Further, SERPIND1 and PON1, both of which were lower among cases, were negatively associated with CRP and inflammatory cytokines (Fig. 3).

Unsupervised clustering analysis using concatenated proteome and cytokine measurements and visualized using principal component analysis (PCA) did not reveal any case-control group separation or clustering (data not shown).

### Cases are characterized by hypoleptinemia

**Figure 4:**
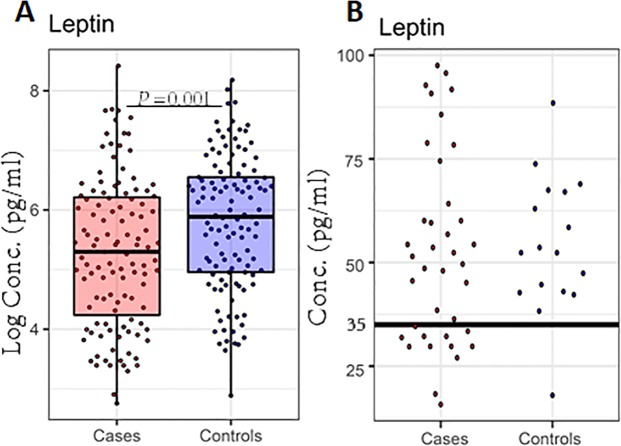
Leptin concentrations are significantly different between cases (n = 119) and controls (n = 119). (**A**) Box plot summary of the median and IQRs of natural logarithms of leptin concentrations. Overlaid dots represent individual data points. (**B**) Leptin concentration, showing only those <100 pg/ml among cases and controls. The dot plot depicts the 35 pg/ml leptin cut-off and dots represent the individual leptin data points <100 pg/ml. P < 0.05 are significant.

Leptin was significantly lower among cases (*P* = 0.001, Fig. 4A-panel Leptin, Table S4). Hypoleptinemia has been previously described as a predictor of mortality in children undergoing treatment for SAM and a leptin cut-off of <35 pg/ml was suggested^29^. A leptin value of <35 pg/ml (n = 13) in the current study also had a high predictive value *P* < 0.0001 (Fig. 4B, data not shown in tables). Additionally, among cases, the median time to death for those with low leptin (<35 pg/ml) was shorter (9 days, IQR; 4.5–16.5) than among those with higher levels (20 days, IQR; 7–38: *P* = 0.04, data not shown in tables). In further analysis, leptin was negatively correlated with inflammatory proteins, von Willebrand factor (VWF) (Fig. 3) and with lower MUAC (*P* < 0.0001; Fig. 3).

### GO term enrichment analysis indicates acute inflammatory response to bacteria

**Figure 5:**
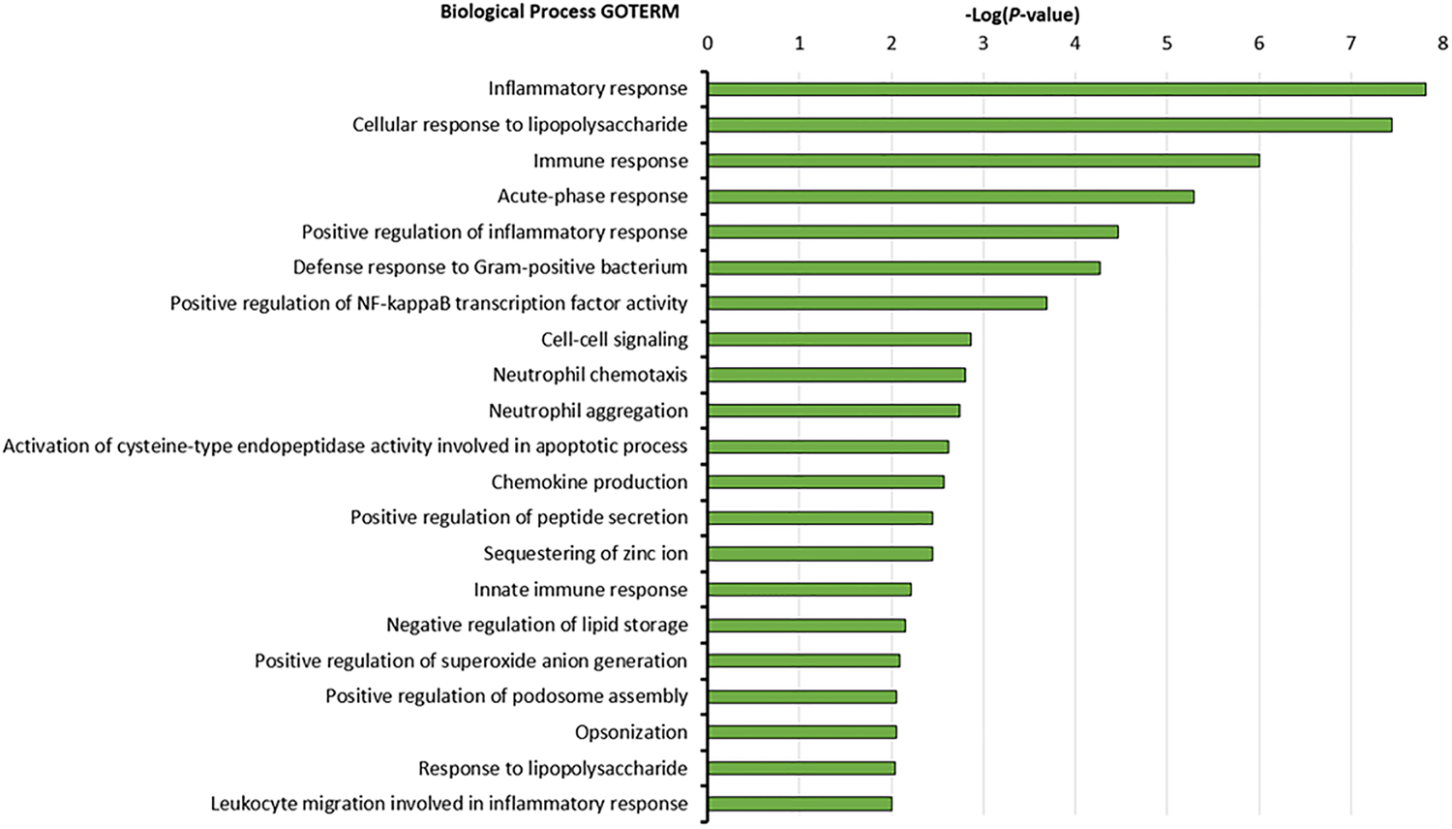
Enrichment analysis of upregulated proteins among cases. Biological processes associated with early post-discharge mortality in children with SAM based on Gene Ontology (GO) analysis. The analysis for pathways enriched was performed using the DAVID Bioinformatics Resources version 6.8. The Bar graphs depict the enriched GO process category and the −log10 of the P value. The P value depicts the probability that a particular biological process is enriched in a group of proteins relative to other biological processes.

Biological process enrichment analysis for proteins upregulated among cases showed enrichment of several Gene Ontology (GO) terms (Fig. 5). These include “inflammatory response”, “cellular response to lipopolysaccharide”, “immune response”, “acute phase response”, “defence response to gram-positive bacteria”, and “positive regulation of nuclear factor-kappa B transcription factor activity” among others (Fig. 5).

### Proteins involved in inflammation and endothelial function are associated with death

We examined the association between individual proteins and mortality by adjusting for putative confounders using multivariate Cox proportional regression, including a term for changes in effect over time (see methods, Table 3). Increased levels of inflammatory proteins including calprotectin, IL8, IL15, IP10, and TNFα were associated with death (Table 3). Elevated plasma levels of VWF and low levels of PON1 and SERPIND1 were also associated with mortality (All *P* < 0.01, Table 3). However, several proteins including CRP, LCP1, Β2M, and G-CSF, that appeared differentially expressed in univariate analysis, were not associated with mortality after adjusting for confounders (See Table 3). A declining effect over time was observed for VWF and inflammatory proteins, IL8 and TNFA, thus children with the highest values died earlier (Table 3; Fig. S1). The effect of age was also noted to interact with time in these models, with the youngest children having higher late mortality risk (all *P* < 0.01), illustrated in Fig. S1.

**Table 3:** Multivariate Cox regression analysis between individual proteins and death.

Measurement	aHR	[95% CI]	*P*	aHR*tvc*	[95% CI]*tvc*	*Ptvc*
CRP	1.32	0.96−1.81	0.087	1.00	0.99−1.01	0.821
S100A8	1.64	1.16−2.33	0.006	0.99	0.98−1.01	0.318
S100A9	1.81	1.21−2.70	0.004	0.99	0.97−1.00	0.105
LCP1	2.37	0.99−5.64	0.052	0.98	0.95−1.02	0.349
LBP	1.73	0.82−3.65	0.149	0.98	0.95−1.01	0.211
VWF	4.19	1.93−9.11	<0.001	0.95	0.93−0.98	0.002
Β2M	2.16	0.93−5.02	0.074	0.99	0.96−1.02	0.321
AGT	2.72	1.23−6.03	0.014	0.98	0.96−1.01	0.269
LRG1	1.21	0.53−2.75	0.646	1.00	0.97−1.03	0.864
SERPINA3	1.57	0.72−3.44	0.262	1.00	0.97−1.03	0.973
PON1	0.39	0.20−0.77	0.007	1.01	0.99−1.04	0.413
SERPIND1	0.38	0.21−0.70	0.002	1.00	0.97−1.03	0.866
GCSF	1.16	0.97−1.39	0.106	1.00	1.00−1.01	0.530
IL8	1.62	1.26−2.08	<0.001	0.99	0.98−1.00	0.016
IL15	1.31	1.05−1.63	0.015	1.00	0.99−1.00	0.149
IP10	1.61	1.16−2.23	0.004	0.98	0.99−1.00	0.203
TNFA	2.20	1.47−3.30	<0.001	0.98	0.97−0.99	0.002
Leptin	0.69	0.52−0.91	0.009	1.01	1.00−1.02	0.121
Total protein	0.48	0.11−2.21	0.348	0.96	0.90−1.02	0.166

Multivariate analysis of log values of protein variables with FDR < 0.05, leptin, and total plasma protein. aHR, Hazard Ratio adjusted for age, sex, nutritional oedema, study site, baseline MUAC, and randomization arm. CI, Confidence Interval; tvc, time varying covariate to test the interaction of each protein covariate with time; HRtvc indicates change in HR per unit time (day). Total protein, total plasma protein.

Plasma leptin was associated with death after adjusting for confounders (*P* = 0.009, Table 3), without evidence of change in effect over time (*P* = 0.1, Table 3). Total plasma protein was not associated with mortality (*P* = 0.35, Table 3).

## Discussion

This study sought to identify biomarkers that suggest biological processes involved in early post-discharge death and that might provide insights into interventional targets to reduce mortality among a group of children at high risk of death. This study focused on early post-discharge mortality (<60 days) because this is the period with the highest mortality rate and is more likely to be related to factors measurable at discharge than later deaths^13,16,18,19^.

Our results show that children who subsequently died had evidence of elevated inflammatory, acute phase, endothelial, and innate cellular responses suggesting exposure to circulating bacteria or bacterial products at a time when they were clinically judged to be stable. Systemic inflammation markers including calprotectin, CRP, and cytokines: G-CSF, IL8, IL15, IP10, and TNFα were increased among cases. Several of these inflammatory markers were independently associated with mortality indicating that mortality in the post-discharge period is related to systemic inflammation. Overall, our data is consistent with other studies showing that during infection, undernourished children have increased positive acute phase proteins (CRP, α1-acid glycoprotein, haptoglobin), IL4, IL6, and IL10 and reductions in IL2, and IFNγ compared to well-nourished children^30^. In addition to heightened inflammation, children with complicated SAM are often characterized by deranged metabolism. For example, the levels of amino acids, plasma protein, non-esterified fatty acids, hormones including leptin, were significantly altered at admission in a Ugandan cohort of children with complicated SAM^29^. Bartz *et al*. showed that inpatient mortality was associated with low levels of high molecular weight adiponectin, hypoleptinemia, as well as high levels of peptide YY and several inflammatory cytokines^29^. Such elevated inflammation and deranged metabolism may partly explain why normal clinical manifestations of infection such as fever or respiratory distress may not be as apparent in children with SAM^11,31^.

Calprotectin is predominantly expressed by neutrophils, monocytes, and activated endothelial and epithelial cells and plays a role in the inflammatory response to LPS by inducing the release of neutrophils from the bone marrow and directing migration to the inflammatory site^32^. It is upregulated in humans and neonatal rats with sepsis^33–35^. Additionally, G-CSF, the principal factor that regulates the maturation, proliferation, and differentiation of neutrophil precursors was elevated among cases but was not associated with mortality after adjusting for nutritional status, age, sex, and other possible confounders. G-CSF also regulates production of TNFα^36^ and promotes the release of IL1Ra^37^. Further, LBP, CRP, and VWF which were observed to be higher among cases, have previously been reported to be elevated in sepsis^38–40^. Interferon-gamma (IFNγ)-inducible protein, IP10, an early mediator of the host response to sepsis^41^ was associated with mortality. IP10 is produced by endothelial cells, monocytes, fibroblasts, and keratinocytes in response to IFNγ and plays an important role in the effector T cell generation and recruitment to sites of inflammation^42^. Our results are consistent with a previous study showing elevated markers of systemic inflammation were significantly associated with inpatient mortality in SAM^43^ and extends those findings to the early post-discharge period. Taken together, these results suggest that among cases who died up to 60 days later, at the time of appearing stable, there was a sepsis-like immunopathogenic profile, likely arising from ongoing active infections that may have been only partially treated or resistant to the antibiotics used, or from circulating translocated bacterial products from loss of intestinal barrier function^44,45^.

Further analysis focused on leptin, an adipose-derived hormone, that acts centrally in the hypothalamus to regulate food intake, body weight, and energy balance^46,47^. Leptin also plays a role in immune homeostasis by differentially regulating the proliferation of naive and memory T cells, enhances Th1 and suppresses Th2 cytokine production, and reverses starvation-induced immunosuppression^48–50^. Very low leptin concentration has previously been reported as a biomarker of mortality in hospitalized Ugandan children undergoing treatment for SAM, and leptin rapidly rises with recovery^29^. In the Ugandan cohort, Mody *et al*. showed that leptin levels were lower in HIV-infected than in non-infected SAM children at diagnosis and that both HIV, hypoleptinemia, and hypoadiponectinemia were associated with increased mortality^51^. Our data shows that leptin was associated with mortality and negatively correlated with inflammation and with lower MUAC which is consistent with findings from inflammatory diseases^13,29,52^. The cut-off of leptin <35 pg/ml suggested by Bartz *et al*., had high positive predictive value but low sensitivity for subsequent death in our study and children with low leptin levels died earlier. Higher overall leptin levels among cases in our study is likely to be because leptin measurements were conducted following medical stabilization and ability to complete therapeutic feeds, whilst the measurements by Bartz *et al*., were conducted before initiation of treatment^29^. It is worth noting that leptin levels are affected by food intake and metabolism, and could be a contributing mediator of the immunosuppressive state in undernutrition^53–55^.

We observed that beside inflammatory markers, higher levels of VWF and AGT were associated with death. VWF is a biomarker of endothelial activation and plays a major role in platelet adhesion and aggregation for haemostasis, and connects haemostatic and inflammatory pathways, recruiting neutrophils and other leucocytes^56–58^. VWF is elevated in patients with inflammatory and metabolic conditions as well as in severe sepsis and septic shock^59^. Our data showed that several inflammatory markers were positively correlated with VWF and is therefore in agreement with previous studies that have demonstrated a link between systemic inflammation and endothelial activation^60–62^. AGT, the common precursor of all angiotensin peptides, is a component of the renin-angiotensin system (RAS), a hormone system that is pivotal to the regulation of blood pressure and homeostasis of water and sodium through actions of angiotensin II^63^. The elevated levels of AGT among children who died may point to fluid and electrolyte disturbances which are often observed in critically ill patients^64^. Taken together, these data may imply that the vascular system and hemodynamic processes in ill undernourished children are perturbed and might not have resolved following in-patient treatment, likely related to ongoing infection.

Further, low levels of PON1 and SERPIND1 were also associated with mortality. PON1 plays a number of roles including as an antioxidant and anti-inflammatory (through destruction of oxidised lipid), helps preserve High Density Lipoprotein function, stimulates cholesterol efflux, and acts as an anti-apoptosis, anti-thrombosis, and anti-adhesion agent^65^. Low levels of PON1 may lead to the dysregulation of the aforementioned processes that are critical for metabolism and general homeostasis thereby increasing risk of mortality in ill undernourished children. Overall, it is likely that inflammation, endothelial activation, and other related biological processes synergistically increase the risk of mortality among malnourished children.

This study was a secondary analysis of samples collected from a previous trial. We acknowledge that the results reported here are hypothesis generating and require validation within additional and larger independent cohorts. The sample size limited main effect estimates, and tests of interaction with time to identifying only larger effects. This study did not include HIV-infected children, whose mortality rates often exceed children without HIV infection. Mortality in HIV-infected children may be due to different underlying biological processes. Using both untargeted and targeted protein analyses, only a limited number of plasma proteins, cytokines, and chemokines were measured and potentially important proteins occurring at low abundances may have been missed. Cellular component ontology analysis of quantified proteins using DAVID^66^ indicated significant enrichment in proteins present in extracellular exosomes, extracellular space, extracellular region, blood microparticle, extracellular matrix, among others (data not shown) and therefore contamination with cellular material was likely minimal if present. We also did not actively measure nutritional factors, hormones, electrolytes, and metabolites which would have contributed further to the understanding of causal mechanisms to mortality. The limited sensitivity of bacterial culture as well as lack of investigation into viral and other infectious causes and deaths occurring in the community limited our ability to evaluate causes of death.

## Conclusions

To our knowledge, this is the first study to evaluate biomarkers associated with subsequent mortality in children who appear to have been medically and nutritionally stabilized prior to discharge. Children who subsequently died were characterized by acute inflammatory and vascular response compatible with exposure to circulating bacteria or bacterial products, reduced antioxidant defence, together with low total plasma protein and hypoleptinemia. Our results suggest early post-discharge mortality is likely to result from ongoing partially-treated bacterial infections or hazardous intestinal bacterial translocation.

## Subjects and Methods

### Study design and patient recruitment

We conducted a nested case-control study using samples and clinical data obtained at enrolment from a randomized placebo-controlled trial investigating the efficacy of daily co-trimoxazole prophylaxis to prevent long-term mortality among HIV-uninfected children with complicated SAM^19^. Details of the trial protocol including participants inclusion and exclusion criteria have been published^19^. Briefly, the study was conducted in two urban (Mombasa and Nairobi) and two rural (Kilifi and Malindi) hospitals in Kenya and recruited children aged 2 months to 5 years who had been treated and achieving clinically-judged stability according to WHO recommendations. Severe malnutrition was defined as: age 6 months to 5 years; mid upper arm circumference (MUAC) <11.5 cm, age 2 to 6 months; MUAC <11 cm, or kwashiorkor at any age (defined in current WHO guidelines)^19,67^. Children were also HIV rapid test negative; or if under 18 months, HIV-1 PCR negative and no longer breastfeeding for at least 6 weeks.

Children were recruited prior to discharge when they had completed the ‘stabilization’ phase of inpatient care, defined by WHO as not having WHO ‘danger’ or ‘emergency’ signs, improvement in oedema, if present, and able to complete prescribed feeds. Samples and clinical data were collected from study participants prior to initiation of the investigational product: co-trimoxazole or placebo.

For this study, cases (n = 121) were defined as children who died within 60 days while controls (n = 120) were randomly selected children who survived and were not readmitted to hospital during 1 year of follow up. From the original study population of 1778, 147 (8.3%) children died within the first 60 days. Cases and controls were included in this analysis if they had sufficient stored plasma sample for analysis. Differences between included and excluded cases were not statistically significant (Table S1).

### Ethics approval and consent to participate

The study had been reviewed and approved by Kenya Medical Research Institute (KEMRI) Scientific and Ethics Review Committee (SERU: No. 2782) and informed consent had been sought from mother’s or guardians of study participants. All experiments were conducted according to Good Clinical Laboratory Practice guidelines.

### Plasma proteomics

Individual plasma samples (10 μl) were depleted the top 12 abundant plasma proteins using spin columns (Thermo scientific) following manufacturer’s instructions. The volume of the flow through was reduced to ~50 μl and protein concentration determined using Bradford assay (Bio-Rad). Proteins samples (30 μg) were adjusted with 50 mM Triethylamonium bicarbonate (TEAB, Sigma-Aldrich) to 100 μl. The protein solution was then reduced with 40 mM tris(2-carboxyethyl)phosphine (TCEP, Sigma-Aldrich) at 55 °C for 1 hour and subsequently alkylated with 80 mM iodoacetamide (Sigma-Aldrich) for 1 hour protected from light at room temperature. Proteins were precipitated overnight at −20 °C with six volumes of pre-chilled (−20 °C) acetone (Sigma-Aldrich). The samples were then centrifuged at 8,000 g for 10 min at 4 °C and the supernatant discarded.

The acetone-precipitated protein pellet was resuspended in 100 µl of 50 mM TEAB. Trypsin (Sigma-Aldrich) was added to the protein samples at a trypsin-protein sample ratio of 1:15 and protein digestion was allowed to proceed overnight at 37 °C with shaking. A pooled sample was prepared by combining 1 μl aliquot from each sample. Peptide samples were individually labelled using the Tandem Mass Tag (TMT) 10-plex kit (Thermo scientific) according to manufacturer’s instructions. Two isobaric tags were exclusively used to individually label two pooled control samples. The labelled peptides for the 8 individual tags in the 10-plex were subsequently combined to generate individual pools upon which the two common pools were equally distributed. The labelled peptide pools were desalted using P10 C18 pipette ZipTips (Millipore) according to manufacturer’s instructions. Eluted peptides were dried in a Speedvac concentrator (Thermo Scientific) and re-suspended in 15 μl loading solvent (97.05% H_2_O, 2% acetonitrile, 0.05% formic acid).

Peptides (8 μl) were loaded onto a Dionex Ultimate 3000 nano-flow ultra-high-pressure liquid chromatography system (Thermo Scientific) with a 75 µm × 2 cm C18 trap column (Thermo Scientific) and separated on a 75 µm × 25 cm C18 reverse-phase analytical column (Thermo Scientific) at 40 °C. Elution was carried out with mobile phase B (80% acetonitrile with 0.1% formic acid) gradient (2 to 35% B) over 310 min at a flow rate of 0.3 μl/min. Peptides were measured using a Q Exactive Orbitrap mass spectrometer (Thermo Scientific) coupled to the chromatography system via a nano-electrospray ion source (Thermo Scientific). The MS^1^ settings were: Resolution, 70000; Automatic gain control (AGC) target, 3e6; maximum injection time, 120 ms; scan range, 380–2000 m/z; while the MS^2^ settings were: Resolution, 35000; AGC target, 5e4; maximum injection time, 120 ms; isolation window, 2.0 m/z. The top 15 most intense ions were selected for MS^2^ and fragmented with higher-energy collision fragmentation using normalized collision energy of 28 V and these ions were subsequently excluded for the next 30 s.

### Mass spectrometry protein identification and quantitation

Mass spectrometer raw files were analysed by MaxQuant software version 1.6.0.1^68^ and peptide lists were searched against the human Uniprot FASTA database (Downloaded February 2014) using the Andromeda search engine^69^. Cysteine carbamidomethylation and TMT-10plex labelled N-terminus and lysine were set as a fixed modification and N-terminal acetylation and methionine oxidations as variable modifications. The false discovery rate (FDR^70^) was set to 0.01 for both proteins and peptides with a minimum length of seven amino acids and was determined by searching a reverse database. Enzyme specificity was set as C-terminal to arginine and lysine with trypsin as the protease. A maximum of two missed cleavages were allowed in the database search. Peptide identification was performed with an allowed initial precursor mass deviation of up to 7 parts per million (ppm) and an allowed fragment mass deviation of up to 20 ppm. Other parameters were used as default settings for Orbitrap-type data. The 10-plex corrected reporter ion intensity matrix was extracted from the MaxQuant proteingroups output file and batch corrected using the pooled sample channels. Protein groups are clusters of proteins or protein isoforms with high sequence similarity cannot be unambiguously identified by unique peptides (but have only shared peptides) are grouped in one protein group and quantified together. Potential contaminants, protein hits from decoy database, and proteins initially depleted but subsequently detected were excluded before exporting the proteingroup matrix file into the analytical and manipulation environment.

### Total protein, sCD14, Leptin, and cytokines measurement in plasma

Total plasma protein concentration was determined using the bicinchoninic acid assay (BCA assay; Thermo Scientific) according to manufacturer’s instructions using diluted plasma samples (1:50). Human Soluble CD14 (sCD14) and leptin ELISAs (Quantikine R&D systems) were conducted according to manufacturer’s protocols and plates were read on a synergy 4 (BioTek) plate reader. Plasma samples were diluted at 1:200 for sCD14 and 1:10 for leptin. Samples and standards were assayed in duplicate to obtain an average of the absorbance and the concentration of the samples was calculated based on the calibration curves and dilution factor. Cytokine and chemokines (n = 29) concentration in plasma were determined by using a human cytokine magnetic bead assay (EMD Millipore) on the Magpix with Xponent software (version 4.2; Luminex Corp) and acquired Median Fluorescent Intensity data analysed using the Milliplex analyst software (version 3.5.5.0 standard). Table S2 lists all cytokines assessed.

### Bioinformatics and statistical analysis

R statistical software version 3.4.2^71^ and Stata software version 13.1 (StataCorp. LLC USA) were used for analysis. For proteomics, columns containing the protein identifiers (IDs), protein names, gene names, and corrected reporter ion intensity in the protein group matrix file from MaxQuant was batch corrected using control reporter ion intensity channels and log normalised. Except for proteomic measurements, data is presented as medians with interquartile ranges (IQRs), means ± the standard deviations (SD), and percentage.

The Gene ontology (GO) enriched biological processes (BP) of differentially expressed proteins (DEP) and cytokines was determined using The Database for Annotation, Visualization and Integrated Discovery (DAVID) v6.8 Bioinformatics Resource^66^. Homo sapiens was used as “background” for enrichment calculation. Pearson’s pairwise correlation analysis between variables was conducted in Stata.

Differential protein expression analysis for proteomics data was initially carried out using the edgeR toolbox as implemented in R while Two-sample Wilcoxon rank-sum (Mann-Whitney) test was used for the luminex data. The Benjamini and Hochberg (BH) FDR method was used to correct for multiple testing of proteins^70^. To assess variation and determine case-control group separation, exploratory unsupervised clustering using principal component analysis (PCA) and k-means clustering was used.

To examine differences in characteristics between cases and controls, 2-sample *t* test and Wilcoxon rank-sum test (of non-normally distributed variables) were used. Chi-square or Fisher’s exact tests were used to assess differences in proportions. Length-for-age was calculated using the WHO 2006 children growth standard reference. MUAC and length-for-age are presented as z scores ± SD.

To investigate their association with death, proteins with FDR-corrected significant differences between cases and controls were taken into a multivariable Cox regression model in order to adjust for potential confounders (age, sex, MUAC, the presence of oedema, site, and randomised arm). We hypothesised that proteins measured at baseline would have their strongest effect on early deaths, in which case covariates may not have met the proportional hazards assumption for Cox regression. This was confirmed to be true for some protein covariates by examining Schoenfeld residuals. We therefore included a term for an interaction between each protein covariate and time. Age was also found to have an interaction with time and a term for this was also included in each model (illustrated in Fig. S1). The final models yielded two hazards ratios (HR), one for the effect at time = 0 and one for the change in HR per unit time^72^.

### Ethics approval and consent to participate

The study had been approved by Kenya Medical Research Institute (KEMRI) Scientific and Ethics Review Committee (SERU: 2782) and informed consent sought from mothers or guardians of study participants.

## Supplementary information

Supplementary file

## Data Availability

The mass spectrometry raw files generated and analysed in the current study have been deposited to the ProteomeXchange Consortium^73^ (PXD010668), via the MassIVE partner repository (MSV000082745), under the following title: Biomarkers of post-stabilization mortality in severe acute malnutrition.
