# Supplementary material for: Biomarkers of post-discharge mortality among children with complicated severe acute malnutrition
Source: Sci Rep. 2019 Apr 12;9:5981. doi: 10.1038/s41598-019-42436-y (PMC6461700; doi:10.1038/s41598-019-42436-y)
Supplement: Supplementary file 1 — Supplementary file [file 41598_2019_42436_MOESM1_ESM.pdf]

Biomarkers of post-discharge mortality among children with complicated severe acute malnutrition

James M. Njunge[1,2*], Agnes Gwela[1,2], Nelson Kibinge[1], Moses Ngari[1,2], Lydia Nyamako[1], Emily Nyatichi[1,2], Johnstone Thitiri[1,2], Gerard Bryan Gonzales[3], Robert H.J. Bandsma[1,7], Judd L. Walson[1,6], Evelyn N. Gitau[4], and James A. Berkley[1,2,5]

Author affiliations

1. The Childhood Acute Illness & Nutrition (CHAIN) Network, Nairobi, Kenya
2. KEMRI/Wellcome Trust Research Programme, Kilifi, Kenya
3. Department of Gastroenterology, Faculty of Medicine and Health Sciences, Ghent University, Ghent, Belgium
4. African Population and Health Research Centre, Nairobi, Kenya
5. Centre for Tropical Medicine & Global Health, Nuffield Department of Medicine, University of Oxford, Oxford, UK
6. Departments of Global Health, Medicine, Paediatrics and Epidemiology, University of Washington, Seattle, Washington, USA
7. Centre for Global Child Health, The Hospital for Sick Children, Toronto, Ontario, Canada

Correspondence to James M. Njunge, KEMRI/Wellcome Trust Research Programme, PO Box 230-80108, Kilifi, Kenya. E-mail: jnjunge@kemri-wellcome.org

Characteristic	Included (N=121)	Excluded (N=26)	*P*
Gender (female) – No. (%)	59 (49)	15 (58)	0.41
Age (months) – median (IQR)	9 (5-14)	6.5 (4-10)	0.06
Under six months) – No. (%)	34 (28)	9 (35)	0.51
Recruitment site			
Kilifi – no. (%)	5 (4.1)	2 (7.7)	0.50
Mombasa – no. (%)	64 (53)	10 (38)	
Malindi – no. (%)	23 (19)	5 (19)	
Mbagathi – no. (%)	29 (24)	9 (35)	
Clinical Variables			
Nutritional oedema – no. (%)	20 (17)	2 (7.7)	0.25
Mid upper arm circumference – cm ± sd	10.1 ± 1.2	10.2 ± 1.1	0.63
Length-for-age z score ± sd	-3.0 ± 2.0	-3.4 ± 1.8	0.28
Diagnosis at index admission			
Severe pneumonia – no. (%)	49 (41)	16 (62)	0.05
Diarrhoea – no. (%)	68 (56)	12 (46)	0.35
Shock – no. (%)	12 (9.9)	4 (15)	0.42
Clinical signs of rickets — no. (%)	24 (20)	5 (19)	0.94
Known tuberculosis – no. (%)	7 (5.8)	1 (3.9)	0.69
Cerebral palsy – no. (%)	4 (3.3)	0	0.38
Randomized to co-trimoxazole prophylaxis – no. (%)	60 (50)	14 (54)	0.69
Full blood count			
Haemoglobin g/dl ± sd	9.6 ± 2.1	9.4 ± 1.7	0.62
Platelets counts ($x10^3$/L) – median (IQR)	387 (203-531)	358 (136-518)	0.49
WBC counts ($x10^3$/L) – median (IQR)	10.5 (7.9-14.8)	11.7 (9.2-16.7)	0.19
Lymphocytes counts ($x10^3$/L) – median (IQR)	4.7 (3-7.0)	5.8 (4.3-9.3)	0.05
Neutrophils counts ($x10^3$/L) – median (IQR)	3.5 (2.2-5.9)	4.1 (2.9-5.6)	0.73

IQR = interquartile range, sd = Standard deviation, no = number.

Table S1: Baseline characteristics of included and excluded cases (deaths).

	Protein Alias	Name	Official Symbol
1	EGF	epidermal growth factor	EGF
2	Eotaxin	chemokine (C-C motif) ligand 11	CCL11
3	GCSF	colony stimulating factor 3 (granulocyte)	CSF3
4	GMCSF	colony stimulating factor 2 (granulocyte-macrophage)	CSF2
5	IFNα2	interferon, alpha 2	IFNA2
6	IFNγ	interferon, gamma	IFNG
7	IL10	interleukin 10	IL10
8	IL12p40	interleukin 12B (40kDa subunit of IL12)	IL12B
9	IL12p70	interleukin 12 (active heterodimer of IL12A and IL12B)	IL12A and B
10	IL13	interleukin 13	IL13
11	IL15	interleukin 15	IL15
12	IL17A	interleukin 17A	IL17A
13	IL1RA	interleukin 1 receptor antagonist	IL1RN
14	IL1α	interleukin 1, alpha	IL1A
15	IL1β	interleukin 1, beta	IL1B
16	IL2	interleukin 2	IL2
17	IL3	interleukin 3	IL3
18	IL4	interleukin 4	IL4
19	IL5	interleukin 5	IL5
20	IL6	interleukin 6	IL6
21	IL7	interleukin 7	IL7
22	IL8	interleukin 8	IL8
23	IP10	chemokine (C-X-C motif) ligand 10	IP10
24	MCP1	chemokine (C-C motif) ligand 2	CCL2
25	MIP1α	chemokine (C-C motif) ligand 3	CCL3
26	MIP1β	chemokine (C-C motif) ligand 4	CCL4
27	TNFα	tumor necrosis factor	TNF
28	TNFβ	lymphotoxin alpha	LTA
29	VEGF	vascular endothelial growth factor	VEGF

Table S2: Cytokines and chemokines measured.

**Measurement**	*K. pneumoniae*	*P. aeruginosa*	*Non-typhoidal Salmonella* sp.	*S. pneumoniae*
Co-trimoxazole	R		R	R
Amikacin	S		S	
Cefoxitin	S		S	
Ceftazidime	R	R	R	
Augmentin	R		R	
Ampicillin	R		R	
Cefuroxime				
Cefotaxime	R		R	
Chloramphenicol	S		R	S
Ciprofloxacin	R	S	R	
Erythromycin				S
Gentamicin	R	S	R	
Tetracycline				R
Imipenem	S	S	S	
Vancomycin				S
Oxacillin				R
Colistin		S		

R=resistant, S=susceptible

Table S3: Antibiotic sensitivity to bacterial isolates obtained from positive blood cultures after enrolment into the trial.

Measurement	Median (IQR)	Median (IQR)	Median (IQR)	P
	All (n=219)	Cases (n=110)	Controls (n=109)	
Total Plasma Protein (mg/ml)	64 (56.1-72.7)	59.9 (51.4-70)	67.3 (60.1-74.8)	<0.001
	All (n=219)	Cases (n=115)	Controls (n=117)	
Leptin	265.6 (109.6-610.1)	200 (64.2-507)	360 (142-700)	0.0013
	All (n=238)	Cases (n=119)	Controls (n=119)	
sCD14 (mg/ml)	1.7 (1.4-2)	1.8 (1.4-2.1)	1.6 (1.4-1.9)	0.09

Data are presented as median (interquartile range). *P* values were determined using the two-sample Wilcoxon rank-sum (Mann-Whitney) test.

Table S4: Comparison of total protein, sCD14 (soluble CD14), and Leptin concentrations between cases and controls.

Uniprot Accession	Protein names	Gene	P	FDR	LogFC
P05109	Protein S100-A8	S100A8	<0.01	<0.01	-0.9
P02741	C-reactive protein	CRP	<0.01	<0.01	-0.99
P06702	Protein S100-A9	S100A9	<0.01	<0.01	-0.64
P13796	Plastin-2/ Lymphocyte cytosolic protein 1	LCP1	<0.01	<0.01	-0.39
P01019	Angiotensinogen	AGT	<0.01	0.01	-0.25
P18428	Lipopolysaccharide-binding protein	LBP	<0.01	0.01	-0.35
P05546	Heparin cofactor 2	SERPIND1	<0.01	0.01	0.24
F5H6I0	Beta-2-microglobulin	B2M	<0.01	0.01	-0.29
P04275	von Willebrand factor	VWF	<0.01	0.01	-0.34
P02750	Leucine-rich alpha-2-glycoprotein	LRG1	<0.01	0.01	-0.24
P01011	Alpha-1-antichymotrypsin	SERPINA3	<0.01	0.02	-0.23
P27169	Serum paraoxonase/arylesterase 1	PON1	<0.01	0.05	0.2
Q9Y5Y7[ɤ]	Lymphatic vessel endothelial hyaluronic acid receptor 1	LYVE1	<0.01	0.12	-0.53
P22352[ɤ]	Glutathione peroxidase 3	GPX3	0.01	0.47	-0.21
P06727[ɤ]	Apolipoprotein A-IV	APOA4	0.02	0.61	-0.19
P02775[ɤ]	Platelet basic protein	PPBP	0.02	0.7	0.19
O95445[ɤ]	Apolipoprotein M	APOM	0.03	0.75	0.19
H0YAC1[ɤ]	Plasma kallikrein	KLKB1	0.03	0.77	0.14
K7ERI9[ɤ]	Apolipoprotein C-I	APOC1	0.03	0.82	0.21
O14791[ɤ]	Apolipoprotein L1	APOL1	0.04	0.87	0.16
P69891[ɤ]	Hemoglobin subunit gamma-1	HBG1	0.04	0.9	-0.36

The comparison between cases and controls of plasma proteins quantities obtained via LC-MS/MS followed by tandem mass tag quantification revealed 21 differentially expressed proteins ($P<0.05$) among which 12 had false discovery rate (FDR) ≤ 0.05. The fold change is expressed as log10 with negative (-) sign depicting proteins with high expression among cases. [ɤ] Indicates proteins that had a $P <0.05$ but $FDR >0.05$.

Table S5: Differentially expressed plasma proteins among cases and controls.

Measurement	All (n=225)	Cases (n=112)	Controls (n=113)		
Cytokines (pg/ml)	Median (IQR)	Median (IQR)	Median (IQR)	P	FDR
G-CSF	56.5 (25.9-108)	78.9 (34-150)	42.5 (17.3-76.6)	<0.001	0.003
IL8	33.4 (17.6-69.3)	40.9 (21.8-103.5)	27.8 (15.2-50.9)	0.001	0.009
IL15	4.4 (2-9.6)	6.5 (2.4-11.7)	3.4 (1.8-7.4)	0.001	0.009
IP10	985 (582-1855)	1206.5 (669-2790.5)	881 (544-1314)	0.004	0.03
TNFα	51.3 (34.5-68)	56.9 (37.2-79.7)	44.6 (31.2-59.8)	0.005	0.03
IL1RA	114 (25.9-422)	167 (40.6-480)	86.8 (16.8-402)	0.01	0.05
IL5	1.8 (0-4.2)	1.4 (0-3.4)	2.1 (1-6.1)	0.02	0.08
EGF	124 (45-337)	117.5 (38.7-253)	146 (55.6-421)	0.07	0.25
IL6	9.8 (0-88.7)	14.5 (0-100)	6 (0-60)	0.2	0.41
IL10	27.7 (14.6-48.8)	33.4 (13.3-61.8)	24.5 (14.8-43.1)	0.2	0.41
IL13	0 (0-172)	0 (0-156)	0 (0-236)	0.2	0.41
MCP1	210 (139-324)	216.5 (125-390)	203 (142-289)	0.2	0.41
TNFβ	0 (0-152)	0 (0-134)	0.6 (0-195)	0.2	0.41
VEGF	129 (51.1-294)	127.5 (41.5-253)	129 (63-316)	0.2	0.41
IL7	12.2 (6.3-23.5)	10.3 (5.5-23.2)	14.6 (7.9-24.2)	0.3	0.54
IL12p70	3 (0-6.4)	3.1 (1.7-6.9)	3 (0-6)	0.3	0.54
IL4	24.2 (10.4-44.8)	24.2 (10.7-41.7)	10.2 (10.2-47.8)	0.4	0.61
IL17α	5.2 (3-8.2)	5.3 (3-8.6)	4.9 (3-7.6)	0.4	0.61
Eotaxin	130 (89.2-205)	128 (87.6-184)	140 (92.5-206)	0.4	0.61
IL1α	21 (2.6-82.6)	23.8 (3.8-92)	18.3 (1.3-69.8)	0.5	0.69
IL3	3.4 (1.2-7.2)	3.1 (0.4-8.2)	4.3 (1.7-6.8)	0.5	0.69
IFNγ	14.5 (8.8-26.5)	14.8 (8.8-31.8)	14.3 (8.8-25.2)	0.6	0.76
MIP1α	13.1 (5.7-33.6)	13.9 (6.1-13.6)	12.4 (5.5-28.9)	0.6	0.76
IL1b	2.8 (1.6-5.3)	1.6 (2.9-5.9)	2.8 (1.6-4.9)	0.7	0.81
IL2	1.8 (0-3.3)	1.9 (0.7-3.5)	1.7 (0-3.2)	0.7	0.81
IL12p40	13.2 (0-45.4)	13.5 (0-44.9)	13.2 (0-47)	0.8	0.89
IFNα2	62.5 (27.6-121)	61.1 (24.8-138.5)	62.5 (31.3-108)	0.9	0.97
GM-CSF	12.9 (5.7-28.1)	12.8 (6.5-27.5)	13.1 (5.7-29)	1	1
MIP1β	51.6 (36.5-71.6)	52.3 (32.7-74.6)	51.3 (37.9-67.5)	1	1

The comparison between cases and controls of cytokine and chemokine concentrations as determined by a human cytokine magnetic bead assay in plasma among cases and controls. Data are presented as median (interquartile range). P value was determined using the Two-sample Wilcoxon rank-sum (Mann-Whitney) test. * depicts FDR value <0.05.

Table S6: Comparison of plasma cytokines and chemokines concentrations between cases and controls.

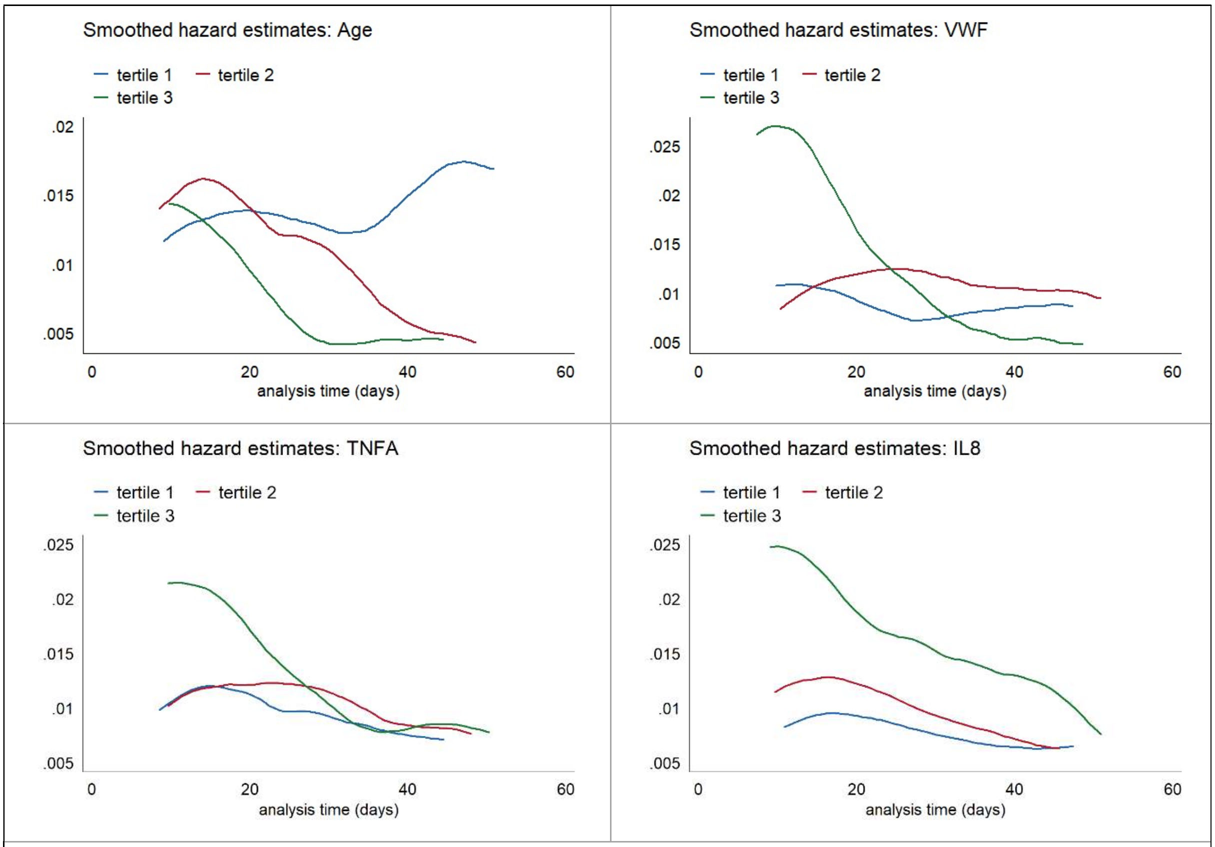

Figure S1: Smoothed hazard estimate curves for time varying covariates; age, VWF, CRP, IL8, and TNFA in the multivariable Cox regression model adjusted for potential confounders (age, sex, MUAC, the presence of oedema, site, and randomised arm). Tertile 1, lowest tertile; tertile 2, middle tertile, tertile 3, highest tertile.
